# On the Influence of Viscoelastic Modeling in Fluid Flow Simulations of Gum Acrylonitrile Butadiene Rubber

**DOI:** 10.3390/polym13142323

**Published:** 2021-07-15

**Authors:** Sebastian Stieger, Evan Mitsoulis, Matthias Walluch, Catharina Ebner, Roman Christopher Kerschbaumer, Matthias Haselmann, Mehdi Mostafaiyan, Markus Kämpfe, Ines Kühnert, Sven Wießner, Walter Friesenbichler

**Affiliations:** 1 Institute of Injection Moulding of Polymers, Montanuniversitaet Leoben, Otto Gloeckel-Straße 2, 8700 Leoben, Austria; walter.friesenbichler@unileoben.ac.at; 2 School of Mining Engineering and Metallurgy, National Technical University of Athens, 157 80 Zografou, Greece; mitsouli@metal.ntua.gr; 3 Anton Paar GmbH, Anton-Paar Straße 20, 8054 Graz, Austria; matthias.walluch@anton-paar.com; 4 Institute of Chemistry of Polymeric Materials, Montanuniversitaet Leoben, Otto Gloeckel-Straße 2, 8700 Leoben, Austria; catharina.ebner@unileoben.ac.at; 5 Polymer Competence Center Leoben GmbH, Roseggerstrasse 12, 8700 Leoben, Austria; roman.kerschbaumer@pccl.at (R.C.K.); matthias.haselmann@pccl.at (M.H.); 6 Leibniz-Institut für Polymerforschung Dresden e.V., Hohe Straße 6, 01069 Dresden, Germany; mostafaiyan@ipfdd.de (M.M.); kaempfe@ipfdd.de (M.K.); kuehnert@ipfdd.de (I.K.); wiessner@ipfdd.de (S.W.); 7 Institute of Materials Science, Technische Universität Dresden, 01062 Dresden, Germany

**Keywords:** rubber rheology, viscoelastic modeling, computational rheology, K-BKZ model

## Abstract

Computational fluid dynamics (CFD) simulation is an important tool as it enables engineers to study different design options without a time-consuming experimental workload. However, the prediction accuracy of any CFD simulation depends upon the set boundary conditions and upon the applied rheological constitutive equation. In the present study the viscoelastic nature of an unfilled gum acrylonitrile butadiene rubber (NBR) is considered by applying the integral and time-dependent Kaye–Bernstein–Kearsley–Zapas (K-BKZ) rheological model. First, exhaustive testing is carried out in the linear viscoelastic (LVE) and non-LVE deformation range including small amplitude oscillatory shear (SAOS) as well as high pressure capillary rheometer (HPCR) tests. Next, three abrupt capillary dies and one tapered orifice die are modeled in Ansys POLYFLOW. The pressure prediction accuracy of the K-BKZ/Wagner model was found to be excellent and insensitive to the applied normal force in SAOS testing as well as to the relation of first and second normal stress differences, provided that damping parameters are fitted to steady-state rheological data. Moreover, the crucial importance of viscoelastic modeling is proven for rubber materials, as two generalized Newtonian fluid (GNF) flow models severely underestimate measured pressure data, especially in contraction flow-dominated geometries.

## 1. Introduction

In order to solve macroscale flow problems like mold filling or extrusion, use is made of continuum mechanics by omitting microscopic discontinuities of the investigated fluid. Discretizing the area of interest by an appropriate mesh, conservation equations of mass, momentum, and energy, are applied, leading to a set of partial differential equations, which are typically solved by means of computational fluid dynamics (CFD) simulation. The prediction accuracy depends upon the set boundary conditions and upon the applied rheological constitutive equation. In most CFD simulations, the viscoelastic nature of polymers is not taken into account. Instead, generalized Newtonian fluid (GNF) flow models are used to describe the relation between the stress and rate of deformation tensors. However, these models fail to reflect important rheological properties (e.g., normal stress differences or transient data). Moreover, they intrinsically assume a ratio of three (Trouton ratio) between steady-state shear and uniaxial elongational viscosities. As a result, they fail to predict extrudate (die) swells or inlet vortices and massively underestimate pressure drops in contraction flow areas [1,2]. Hence, developing and improving viscoelastic constitutive equations is an eminent subject in polymer rheology research [3].

Numerous differential [4,5,6,7,8] and integral [9,10,11,12] models have been proposed to describe the viscoelastic behavior of polymeric fluids. Most of them are based on either a network [7,9,12] or reptation (tube) [6,13] theory. Although some differential equations such as the (extended) Pom-Pom [14] or exponential Phan Thien-Tanner (ePTT) [15] model are in a good agreement with rheological data, a conflict of interest arises as follows: An accurate description of a real polymer melt requires a multi-mode formulation. Each added number of mode increases the degrees of freedom (dof) and therefore the complexity from a numerical point of view [2].

The second group of models overcomes this problem by applying an integral formulation [2]. Inspired by the theory of rubber elasticity [11] Kaye [9], Bernstein, Kearsley and Zapas [12] proposed a temporary network model (K-BKZ), which has been continuously improved over the past 50 years [2,16]. In the current mathematical formulation implemented in Ansys POLYFLOW (Equations (1)–(3) and (6)) it is able to reflect steady-state shear η and steady-state uniaxial elongational $\eta_{e}$ viscosities, first $N_{1}$ and second $N_{2}$ normal stress differences, start-up shear $\eta^{+}$ and start-up uniaxial elongational $\eta_{e}^{+}$ viscosities as well as storage $G^{\prime }$, loss $G^{\prime \prime }$ and relaxation $G\left(t\right)$ moduli of unfilled polymer melts in the linear viscoelastic (LVE) and non-LVE region well [2]:

(1) $$\overset{=}{\tau }=\frac{1}{1-\;\theta }\int_{-\infty }^{t}M\left(t-t^{\prime }\right)H\left(I_{C^{-1}},{\;I}_{C}\right)\;\left[{\overset{=}{C}}_{t}^{-1}\left(t^{\prime }\right)\;+\;\theta \;\cdot \;{\overset{=}{C}}_{t}\left(t^{\prime }\right)\right]dt^{\prime }$$

with the stress tensor $\overset{=}{\tau }$, the material constant θ, the present time t, the past time $t^{\prime }$, the memory function $M\left(t-t^{\prime }\right)$, the damping function $H\left(I_{C^{-1}},{\;I}_{C}\right)$, the Finger strain tensor ${\overset{=}{C}}_{t}^{-1}\left(t^{\prime }\right)$, and the Cauchy–Green tensor ${\overset{=}{C}}_{t}\left(t^{\prime }\right)$. The material constant θ is given by Equation (2):

(2) $$\frac{N_{2}}{N_{1}}=\frac{\theta }{1-\theta }\;$$

In Equation (1) the stress results from an integral over the past time, where effects of strain are separated from those of time. The latter is described through a memory function:

(3) $$M\left(t-t^{\prime }\right)\;=\overset{N}{\underset{i=1}{\sum }}\frac{g_{i}}{\lambda_{i}}\cdot \exp\left(-\frac{t-t^{\prime }}{\lambda_{i}}\right)\;$$

with i representing the ith mode of the total number of modes N, the relaxation moduli $g_{i}$ and relaxation times $\lambda_{i}$. The required relaxation spectrum can be easily obtained from small amplitude oscillatory shear (SAOS) tests and more precisely fitting $G^{\prime }$ and $G^{\prime \prime }$ data (ω is the angular frequency) applying Equations (4) and (5):

(4) $$G^{\prime }\left(\omega \right)\;=\overset{N}{\underset{i=1}{\sum }}g_{i}\;\frac{\omega^{2}{\;\lambda }_{i}{}^{2}}{1+\omega^{2}{\;\lambda }_{i}{}^{2}},$$

(5) $$G^{\prime \prime }\left(\omega \right)\;=\overset{N}{\underset{i=1}{\sum }}g_{i}\;\frac{{\omega \;\lambda }_{i}}{1+\omega^{2}{\;\lambda }_{i}{}^{2}}.\;$$

The dependency of rheological properties on the imposed strain is given by Wagner’s [17,18] damping function:

(6) $$H\left(I_{C^{-1}},{\;I}_{C}\right)\;=\exp(-\alpha \sqrt{{\beta I}_{C^{-1}}+\left(1-\beta \right)I_{C}-3})\;$$

where α and β are fitting parameters to be determined from shear and elongational data and $I_{C^{-1}}$ as well as $I_{C}$ are the first invariants of the Finger strain tensor and Cauchy–Green tensor, respectively.

Recently, different research groups had great success applying K-BKZ equations to reproduce benchmark polymer-flow phenomena such as inlet vortices [19] or extrudate swelling [20,21,22]. Moreover, the K-BKZ model was found to predict measured pressure drops with an accuracy that has not been reported for any other constitutive rheological model [21,23,24]. However, in all aforementioned cases the investigated fluid was an unfilled polyolefin melt.

In our recent studies [25,26] the applicability of the K-BKZ/Wagner model was tested to highly carbon black filled rubber compounds. This class of materials differs distinctively from polyolefin melts, as rubber compounds exhibit a material behavior dominated by elasticity. Moreover, they are processed at significant lower temperature levels (80 °C to 120 °C) and contain reinforcing filler particles. When comparing recorded pressure drops in various capillary dies to K-BKZ/Wagner predictions considerable deviations were observed [25,26]. One possible explanation is that the K-BKZ/Wagner model is not applicable to highly filled polymeric melts. The second possible explanation is that the K-BKZ/Wagner model is not applicable to rubber materials. In order to answer this open research question the present study tests for the first time the ability of the K-BKZ/Wagner model to correctly predict pressure drops of an unfilled gum rubber in CFD simulation.

## 2. Materials and Methods

### 2.1. Material

**Figure A1:**
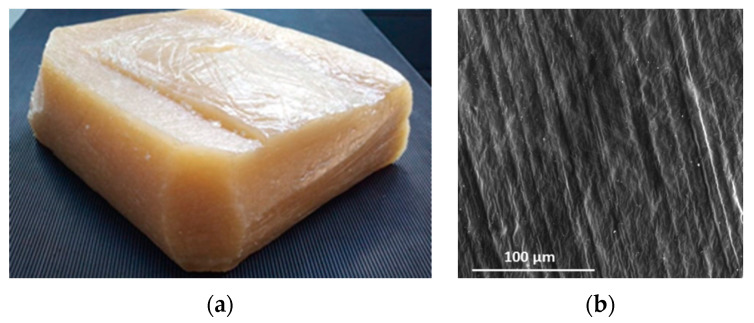
(**a**) Image of gum NBR (PERBUNAN^®^ 3965 F) delivered as bale; (**b**) scanning electron microscope (SEM) image of an exemplary compression molded sample (∅ = 8 mm, thickness = 1 mm) of gum NBR.

The investigated material is a butadiene acrylonitrile copolymer labeled PERBUNAN^®^ 3965 F. This industrial grade was obtained from ARLANXEO (ARLANXEO Deutschland GmbH, Dormagen, Germany) containing no filler particles as well as no curing system (Figure A1). The Mooney viscosity ML (1 + 4) [27] is according to product specifications [28] without pre-treatment 65 ± 7 Mooney units (MUs), the acrylonitrile content is 39 ± 1.0 wt %, and specific gravity 0.99. It was delivered in bales (Figure A1a) and will be referred to as “gum NBR” in the following chapters.

### 2.2. Rheological Testing

#### 2.2.1. SAOS

**Figure A2:**
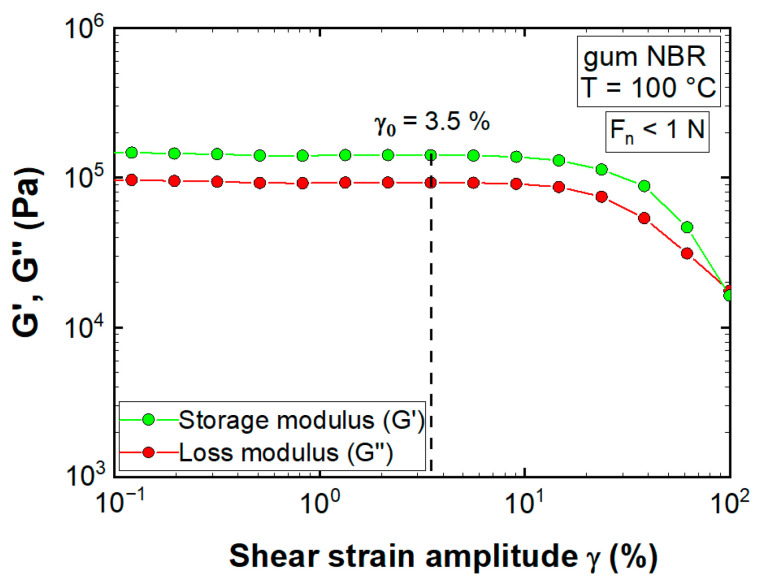
Exemplary amplitude sweep measurement performed with the MCR 501 device at 100 °C to detect the linear viscoelastic deformation range of gum NBR, with the selected shear strain rate of 3.5% for the frequency sweep measurements.

LVE rheological properties were detected performing small amplitude oscillatory shear tests applying the Modular Compact Rheometer (MCR) 501 (Anton Paar GmbH, Graz, Austria) in parallel-plate configuration with serrated surfaces [25]. For all experiments with the MCR 501 device, samples of 25 mm diameter and approximately 1 mm thickness were prepared. As a second measurement device the rotorless Rubber Process Analyzer (RPA) D-MDR 3000 (MonTech Werkstoffprüfmaschinen GmbH, Buchen, Germany) was tested, employing a closed, bi-conical, and grooved measurement chamber. In order to specify the LVE deformation range of gum NBR, first, runs with constant angular frequency and step-wise amplitude increase of the sinusoidal strain (“amplitude sweeps”) were done. One exemplary curve is displayed in Figure A2, where a strain amplitude of $\gamma_{0}$ = 3.5% was selected for further frequency sweep measurements, which is well within the linear viscoelastic deformation range of gum NBR. This procedure was performed for each individual measurement setting. Second, the influence of sample preparation (roller milled vs. compression molded specimens), normal force (F_n_), pre-shearing as well as the influence of the measurement device (MCR 501 vs. RPA) was analyzed by keeping the selected strain amplitude constant and varying the angular frequency level (“frequency sweep”). Third, storage $G^{\prime }$ and loss $G^{\prime \prime }$ moduli were recorded with the MCR 501 device at four different temperature levels (60, 80, 100, and 120 °C) and a master curve was derived by time-temperature shifting (TTS) to the reference temperature of 100 °C. The mastered LVE moduli were subsequently used to fit the memory function in Equation (3).

#### 2.2.2. High Pressure Capillary Rheometry

The pressure-driven flow of gum NBR was studied employing a high pressure capillary rheometer (HPCR) of the type Rheograph 50 (GÖTTFERT Werkstoff-Prüfmaschinen GmbH, Buchen, Germany). In order to analyze possible slippage at the wall, first, tests with two abrupt (entrance angle ϕ = 180°) capillary dies of same length-to-diameter (L/D) ratio (L/D = 10/1, 20/2) were carried out. The entrance pressure loss was measured in both cases with tapered (ϕ = 90°) orifice dies (L/D = 0.2/1, 0.2/2). Second, the pressure linearity in dependence of the capillary length (“Bagley plot”) was analyzed by measuring pressure drops in two additional abrupt capillary dies (L/D = 5/1, 20/1). Third, the steady-state shear viscosity η was calculated for the 1 mm diameter die set applying Bagley and Weißenberg–Rabinowitsch corrections. This material property (η) was used to fit the damping parameter α in Equation (6) as well as GNF models. Finally, Binding’s model [29] was selected to estimate the steady-state uniaxial elongational viscosity $\eta_{e}$ from entrance pressure losses and subsequently used to fit the final damping parameter β in Equation (6). All tests were performed at a reference temperature of 100 °C.

### 2.3. Constitutive and Numerical Modeling

In order to test K-BKZ/Wagner’s applicability to correctly predict pressure drops of gum NBR, the commercial software package Ansys POLYFLOW (ANSYS Inc., Canonsburg, CA, USA) was selected to simulate the pressure-driven flow of the high pressure capillary rheometer (HPCR) experiment.

**Figure 1:**
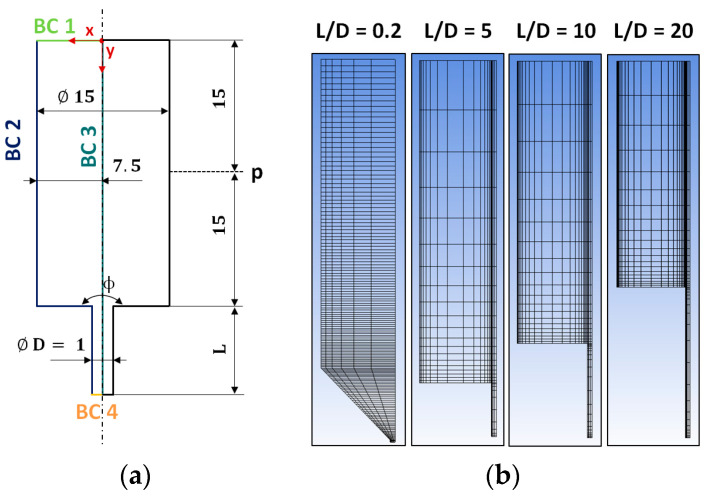
(**a**) Computational fluid dynamic (CFD) simulation setup including boundary conditions (BCs) 1 to 4, dimensions, and position of the pressure transducer (p); (**b**) Meshes for the tapered orifice (L/D = 0.2/1; ϕ = 90°) and three abrupt capillary dies (L/D = 5/1, 10/1, 20/1; ϕ = 180°) utilized in numerical calculations.

A two-dimensional axisymmetric finite element model was built for one tapered orifice die (L/D = 0.2/1) and three abrupt capillary dies (L/D = 5/1, 10/1, 20/1). Figure 1a illustrates a schematic drawing of the numerical setup including boundary conditions (BCs), geometrical dimensions, as well as the position of the pressure evaluation (p), which corresponds to the position of the pressure transducer in the HPCR. The mesh design was based on [20,24], who used a denser grid moving toward the stress singularity at the entrance of the die.

Mesh independency was checked in one of our previous studies [25] by comparing predicted pressure drops using the meshes displayed in Figure 1b to denser ones. The applied meshes in the present study (Figure 1b) consisted of 936 elements (1099 nodes) for the tapered orifice die (L/D = 0.2/1), 450 elements (501 nodes) for the short abrupt capillary die (L/D = 5/1), and 600 elements (666 nodes) for the two remaining abrupt capillary dies (L/D = 10/1, 20/1).

In all calculations steady-state and isothermal flow conditions were assumed leading for an incompressible fluid to the following governing equations:

(7) $$\nabla \;\cdot \;\bar{v}=0,$$

(8) $$-\nabla p+\nabla \;\cdot \;\overset{=}{\tau }=0$$

where $\bar{v}$ is the velocity vector, p is the pressure, and $\overset{=}{\tau }$ is the stress tensor. Effects of inertia and gravity were neglected due to the high viscosity of gum NBR. In case of viscoelastic modeling the stress tensor $\overset{=}{\tau }$ was calculated with Equations (1)–(3) and (6), which represent the K-BKZ/Wagner model. Viscous stresses were computed as:

(9) $$\overset{=}{\tau }=2\;\eta \left(\dot{\gamma }\right)\;\overset{=}{D}$$

where $\overset{=}{D}$ is the rate of deformation tensor, with two different GNF flow models describing the shear dependence of the viscosity $\eta \left(\dot{\gamma }\right)$:

(10) $$\eta \left(\dot{\gamma }\right)=K{\left(\lambda \;\cdot \;\dot{\gamma }\right)}^{n-1}.$$

The power-law model in Equation (10) is arguably the simplest and most common GNF model in rubber rheology with K as the consistency index, λ as the natural time, and n as the power-law index. It assumes a power-law behavior of the steady-state shear viscosity even in the LVE region. However, most polymer melts exhibit a constant viscosity at low shear rates (“Newtonian plateau”). Consequently, a second GNF model was applied able to reflect the aforementioned material behavior properly:

(11) $$\eta \left(\dot{\gamma }\right)\;={\;\eta }_{0}/\left(1+{\left(\lambda \;\cdot \dot{\gamma }\right)}^{m}\right).$$

The Cross model in Equation (11) consists of the three model parameters $\eta_{0}$, λ, and m, which represent the zero-shear-rate viscosity, the natural time, and the Cross-law index (m ≙ 1 −  n), respectively.

Finally, all boundary conditions applied in this study are listed in Table 1 with corresponding surfaces illustrated in Figure 1a.

## 3. Results and Discussion

**Figure 2:**
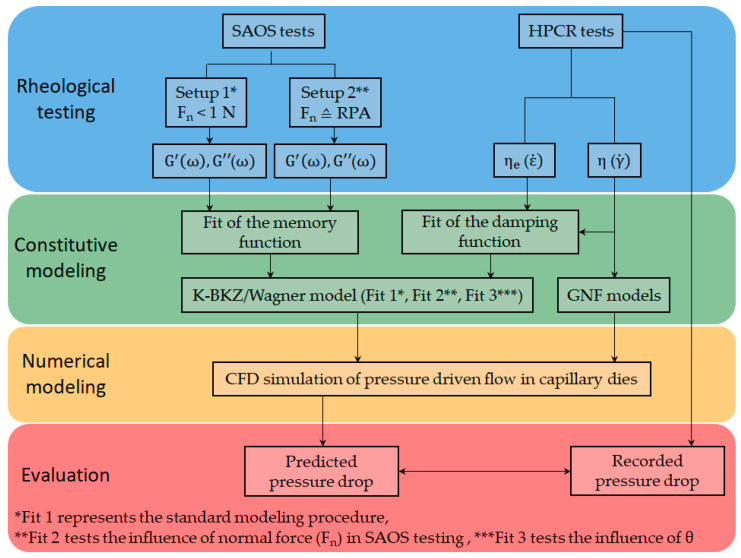
Flow chart displaying the research design of the present study.

The overall goal of the study is to answer the open research question whether the K-BKZ/Wagner model is able to correctly predict pressure drops of an unfilled gum rubber in CFD simulation. In order to address this objective, a specific research approach was designed, which is depicted in Figure 2 in a flow chart schematic.

First, rheological tests are performed in the LVE (SAOS) and non-LVE (HPCR) deformation range of gum NBR. The material properties obtained from these measurements are storage (G’) and loss (G”) moduli, both in dependence of the angular frequency (ω) as well as steady-state shear (η) and uniaxial elongational ($\eta_{e}$) viscosities, which are in dependence of the shear ($\dot{\gamma }$) and strain ($\dot{\varepsilon }$) rate, respectively. These material properties are used to determine the model parameters of the K-BKZ/Wagner, Cross (GNF), and power-law (GNF) models. Additionally, the influence of normal force applied on the sample in SAOS testing as well as the influence of the model parameter θ (Equation (2)) is analyzed by performing in total three K-BKZ fits. Finally, CFD simulations are performed aiming to compare predicted with recorded pressure data of the high pressure capillary rheometer experiment.

### 3.1. Rheological Testing and Constitutive Modeling

**Figure 3:**
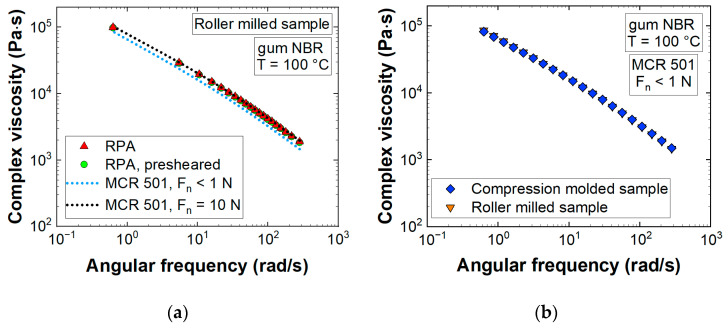
(**a**) Effect of applied normal force (F_n_) on the sample during the measurement and influence of the measurement devices (RPA vs. MCR 501) on the complex viscosity of gum NBR; (**b**) No influence of sample preparation detected on the complex viscosity of gum NBR.

In rubber rheology a testing instrument called Rubber Process Analyzer is widely employed to determine viscoelastic properties before, during, and after curing. This rotational shear rheometer consists of a closed and sealed die system, where the tested rubber is compressed by a pre-defined clamping pressure leading to normal forces on the sample during the measurement. This setup aims to prevent any slippage and differs from conventional dynamic rheometers like the MCR 501 device, where test specifications recommend keeping normal forces at a minimum. Comparing the complex viscosity ($\eta^{*}$) of gum NBR, normal forces applied on the sample did not affect the shape of the curve but shifted measured data to higher values (Figure 3a). Applying a normal force of 10 N using the MCR 501 device, $\eta^{*}$ corresponded to RPA data. On the other hand, neither sample preparation (roller milled vs. compression molded) nor pre-shearing the sample for 240 s with a strain amplitude of 42% and constant angular frequency of 31.42 rad/s (recommended by Fasching [30] for highly filled rubber compounds) had any effect on $\eta^{*}$ (Figure 3). The detected insensitivity of gum NBR validated the listed set of pre-shearing parameters, as they did not damage macromolecular chains and may serve for highly filled rubber compounds as a fixed precondition to break down filler networks and minimize their impact on measured LVE properties [25]. The mean measurement deviations of the four settings displayed in Figure 3 (RPA; RPA, presheared; MCR, F_n_ < 1 N; MCR 501, F_n_ = 10 N) are 0.3, 0.3, 1.8, and 3.4%, respectively. In the present study, both MCR setups (F_n_ < 1 N, F_n_ = 10 N) were further used to determine G’ and G” aiming to fit the memory function in Equation (3) and finally analyze the influence of normal force in SAOS testing to viscoelastic modeling and pressure prediction in CFD simulation.

Next, time-temperature superposition was applied, to extend the frequency range of LVE modeling. From a thermo-rheological point of view, rubber materials are complex fluids, where vertical shifting may be necessary in order to construct a master curve for G’ and G”. Consequently, a guideline presented in [25] was followed, where horizontal shift factors ($a_{t}$) were obtained by mastering first the loss factor (tanδ), which is intrinsically invariant to vertical shifting ($b_{t}$):

(12) $$\tan\delta =\frac{b_{t}\cdot G^{\prime \prime }\left(a_{t}\;\cdot \;\omega \right)}{b_{t}\cdot G^{\prime }\left(a_{t}\;\cdot \;\omega \right)}.$$

**Figure 4:**
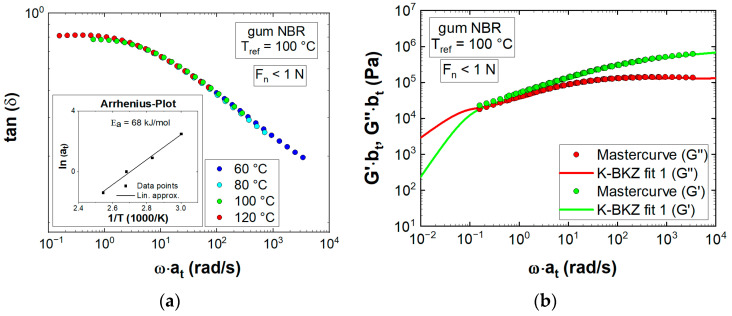
(**a**) Time-temperature superposition of the dissipation factor (tanδ) and corresponding Arrhenius plot; (**b**) K-BKZ model predictions of storage G’ and loss G” moduli compared to experimental data at the reference temperature T_ref_; K-BKZ fit 1 represents LVE data measured at a normal force (F_n_) < 1 N.

Figure 4a displays the mastered dissipation factor at the reference temperature of 100 °C. Moreover, natural logarithms of $a_{t}$ were plotted against the inverse temperature, resulting in the so-called “Arrhenius Plot”.

**Figure A3:**
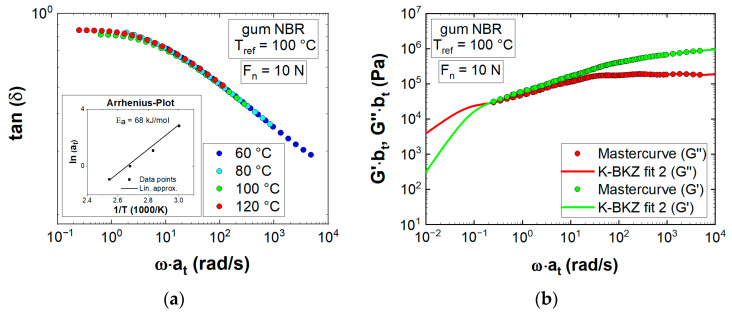
(**a**) Time-temperature superposition of the phase angle δ and corresponding Arrhenius plot; (**b**) K-BKZ model predictions of storage $G^{\prime }$ and loss $G^{\prime \prime }$ moduli compared to experimental data at the reference temperature T_ref_; K-BKZ fit 2 represents fitted LVE data measured at F_n_ = 10 N.

The calculated activation energy (E_a_) of 68 kJ/mol corresponded well with values obtained for NBR and HNBR compounds [25,26] and was insensitive to F_n_ (Figure A3a). Second, vertical shifting was applied to G’ and G’’ measured at 60, 80, and 120 °C, minimizing any deviations to the reference temperature segment (Figure 4b). Over the whole measured angular frequency range, G’ exceeded G” (no crossover point), proving a material response that was dominated by elasticity. Finally, LVE moduli were used to determine relaxation moduli $g_{i}$ of the memory function in Equation (3). A fit was performed, which minimized the sum of squared differences according to Equations (4) and (5), keeping pre-defined relaxation times $\lambda_{i}$ and modes (N = 8) constant. To give the fit a physical meaning, a constraint was added, which ensured that relaxation moduli would not decrease with increasing corresponding relaxation time ($g_{i}$ ≥ $g_{i+1}$). The obtained material parameters (Table A1) well described the LVE material behavior of gum NBR in a frequency range of almost five decades (Figure 4b). The mean measurement deviations of the three recorded properties G’, G”, and tan (δ) were 2.6, 3.1, and 0.6%, respectively.

Next, the exact same procedure was carried out for the second rheological characterization setup (F_n_ = 10 N). Table A2 lists corresponding material parameters and Figure A3b compares model predictions with measured data. The mean measurement deviations of G’, G”, and tan (δ) with an applied normal force of 10 N were 3.9, 3.9, and 0.8%, respectively. In the present study “K-BKZ fit 1” represented LVE data measured according to conventional test specifications (F_n_ < 1 N) and “K-BKZ fit 2” represented LVE data, where a normal force of 10 N was applied on the sample during the measurement. The latter was similar to conditions in a Rubber Process Analyzer for gum NBR.

**Figure 5:**
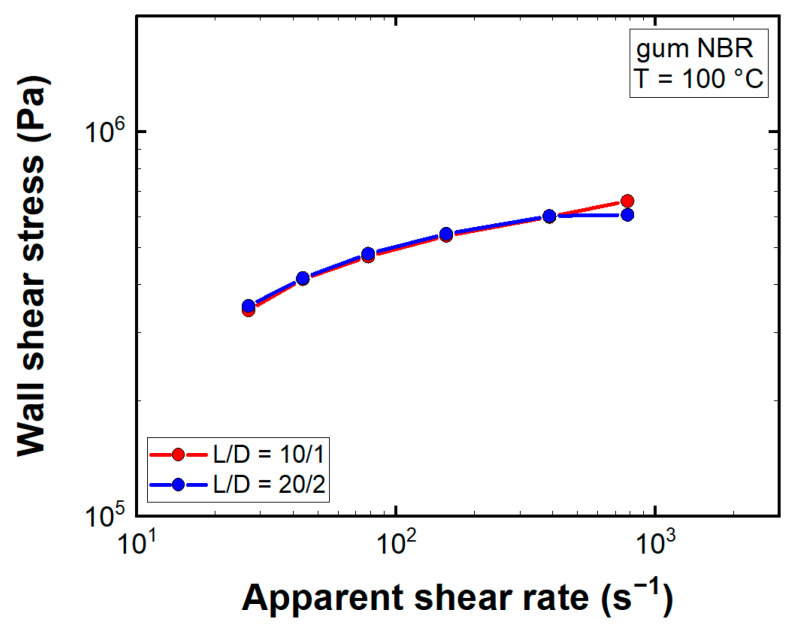
Entrance pressure corrected flow curves of two capillary dies with same length-to-diameter (L/D) ratios but different capillary diameters.

A key assumption in rheological testing is no slip at the wall, which indicates adhesion of the investigated fluid to the capillary die or to the plates, in cases of SAOS testing. However, as reviewed by Hatzikiriakos [31], this classic boundary condition is not always valid for polymer melts, especially at higher wall shear stresses. A popular method for detection was introduced by Mooney [32], who proved that for a wall slipping fluid the observed flow curves become dependent on the diameter of the capillary die. Comparing flow curves recorded with two dies of same L/D ratio but different geometry, gum NBR exhibits no clear dependency on the die diameter (Figure 5).

**Figure A4:**
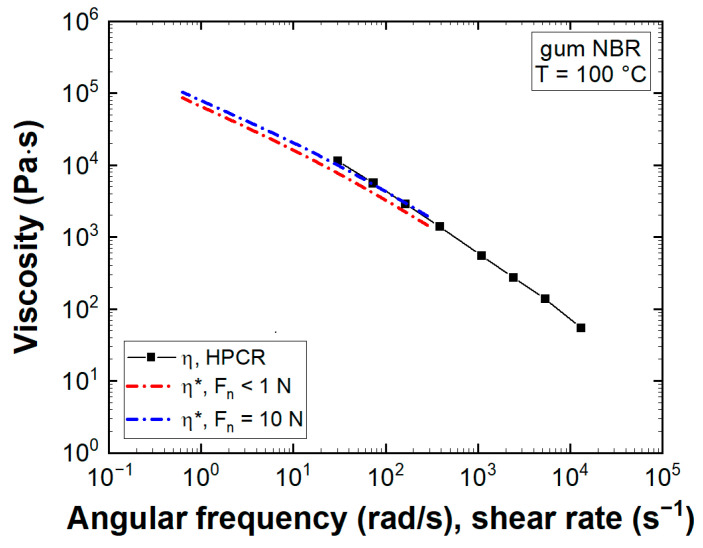
Complex viscosities $\eta^{*}$ compared to steady-state shear viscosity η.

If in fact slip was present in HPCR experiments, one would expect the wall shear stress in the L/D = 20/2 die to be larger than in the L/D = 10/1 die, especially at higher apparent shear rate levels, and the calculated steady-state shear viscosity to be substantially lower than the complex viscosity. Comparing the aforementioned material properties (Figure A4), the steady-state shear viscosity of gum NBR even exceeded the complex viscosity (F_n_ < 1 N). For the second SAOS testing setup, where a normal force of 10 N was applied on the sample during the measurement, the Cox–Merz rule [33] ($\left|\eta^{*}\left(\omega \right)\right|$ = η($\dot{\gamma })\;\left|\omega \;=\dot{\gamma }\right.$) was obeyed. Moreover, at the highest apparent shear rate level the wall shear stress in the L/D = 20/2 capillary die was lower than in the L/D = 10/1 die. These observations indicated no slippage at the wall for gum NBR.

**Figure A5:**
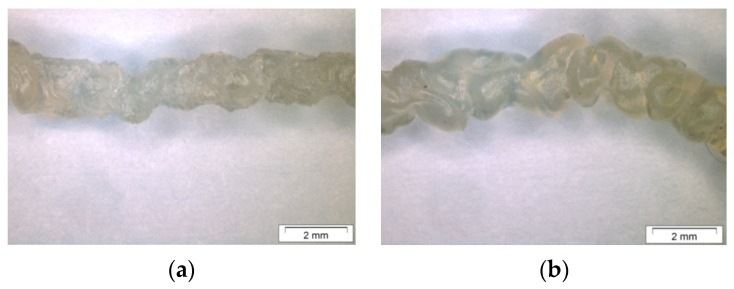
Light microscope images of strands extruded through an abrupt capillary die with a length-to-diameter (L/D) ratio of 10/1 at an apparent shear rate level of (**a**) 27 s^−1^; (**b**) 30906 s^−1^.

Furthermore, strong extrudate swelling was observed even at low apparent shear rate levels (Figure A5). This non-linear flow phenomenon arises from stored energy of uncoiled polymer molecules due to shear and elongational stresses in the capillary die. These uncoiled molecules strive to reach the former state of higher entropy resulting in additional forces that press the fluid in the capillary against the wall and may prevent slippage. Thus, a no slip BC was applied in CFD simulations.

Next, the detected pressure drops of the 1 mm die set were plotted in dependence of the capillary length (Bagley plot [34]), with mean measurement deviations of 1.5, 3.1, 1.1, and 1.4% for the L/D = 0.2, 5, 10, and 20 capillary dies, respectively. The apparent shear rate was calculated applying Equation (13):

(13) $${\dot{\gamma }}_{a}=\frac{32Q}{{\pi D}^{3}}$$

where Q is the volumetric flow rate and D the diameter of the capillary die.

**Figure 6:**
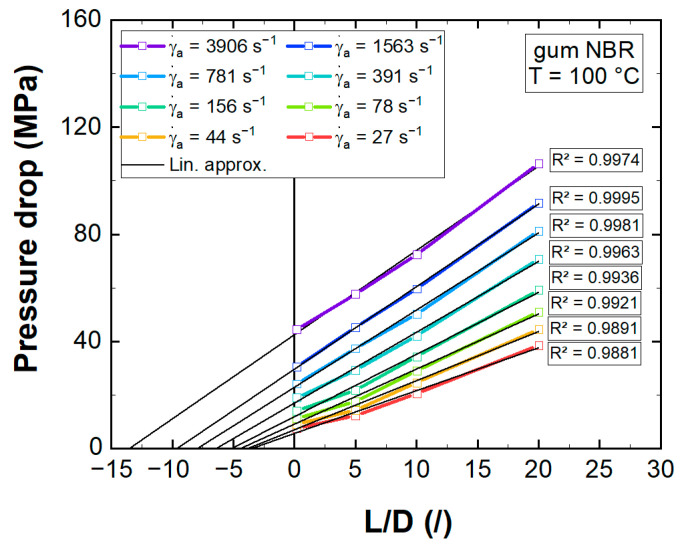
Measured pressure drops for various apparent shear rates (${\dot{\gamma }}_{a}$) in dependence of the capillary die length (Bagley plot) including linear approximations (Lin. approx.) with coefficients of determination (R²).

**Figure A6:**
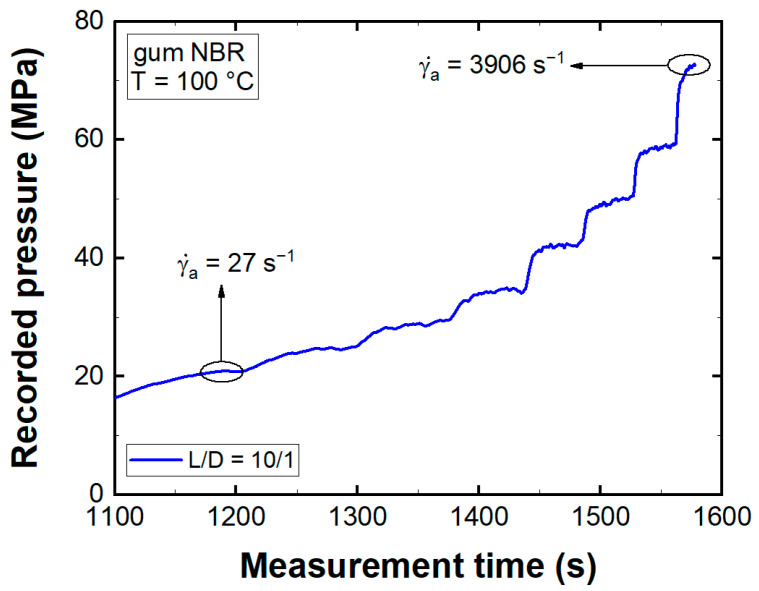
Recorded pressure in the L/D = 10/1 capillary die in dependence of the measurement time with apparent shear rate levels displayed ranging from 27 s^−1^ to 3906 s^−1^.

All apparent shear rate levels exhibited high linearity of the pressure drop, despite some observed pressure fluctuations (Figure A6), with coefficients of determination (R^2^) exceeding 98% (Figure 6).

A highly linear Bagley plot indicated that pressure and temperature dependencies of the viscosity effectively leveled off each other [25,26]. Since Ansys POLYFLOW offers no rheological model, which accounts for the pressure dependency of the viscosity, the best option was to consider neither of them. Consequently, isothermal flow conditions were assumed in CFD simulations, and η was modeled as a simple shear rate dependent function.

Next to the two damping parameters α and β, the material constant θ also needed to be determined to complete the K-BKZ modeling. For most polymeric melts small negative values are reported for θ [35]. Since no experimental data of the second normal stress difference were available for gum NBR, this study tested the sensitivity of θ with respect to pressure prediction in CFD simulation. Thus, a third fit (K-BKZ fit 3) was performed, which maintained the relaxation spectrum of K-BKZ fit 1 and set θ to –0.15 (Table A3). K-BKZ fit 1 (Table A1) and K-BKZ fit 2 (Table A2) both assumed θ to be zero.

In a simple shear flow, Equation (6) depends only on one parameter, so the steady-state shear viscosity was used next to determine α. The final model parameter β was obtained from uniaxial elongational viscosity data calculated from entrance pressure losses according to Binding [29]. In general, extensional rheometers developed by Sentmanat [36], Meissner [37,38], or Münstedt [39] are recommended to characterize the elongational behavior of polymeric melts. However, in the present study we preferred material properties obtained with the HPCR to fit damping parameters α and β, since recorded pressure drops of the exact same measurement device are aimed to be predicted in CFD simulations. All fits were performed in MATLAB minimizing the sum of squared differences to measured data.

**Figure 7:**
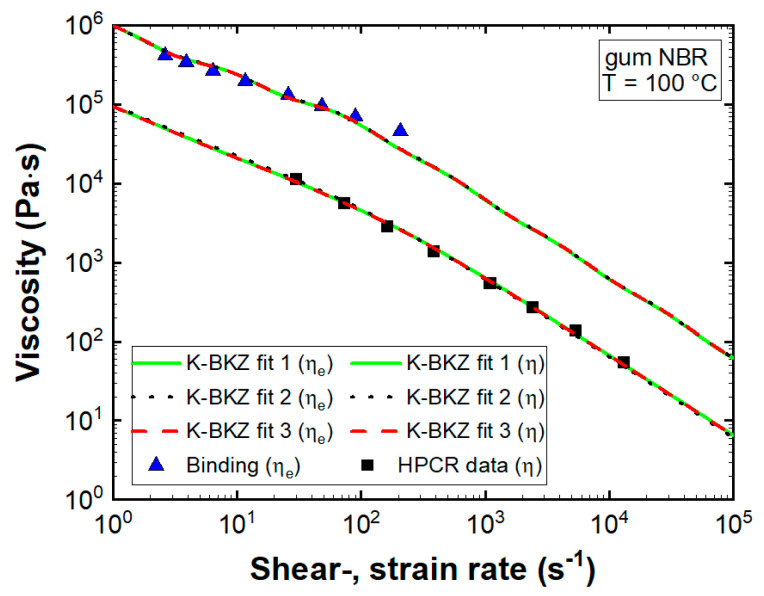
Steady-state shear (η) and uniaxial elongational ($\eta_{e}$) viscosities of gum NBR compared to K-BKZ model predictions; K-BKZ fit 1 represents fitted LVE data measured at F_n_ < 1 N (θ = 0), K-BKZ fit 2 represents fitted LVE data measured at F_n_ = 10 N (θ = 0), and K-BKZ fit 3 represents fitted LVE data measured at F_n_ < 1 N (θ = −0.15).

Despite applying different relaxation spectra (fit 1 vs. fit 2) and different values for the material constant θ (fit 1 vs. fit 3), model predictions of all fits described the steady-state shear viscosity of gum NBR well (Figure 7).

A higher damping parameter α compensated for the vertical shifted LVE data of K-BKZ fit 2 (F_n_ = 10 N). However, all fits overestimated the uniaxial elongational viscosity at low strain rates and underestimated $\eta_{e}$ at higher ones. To improve K-BKZ modeling of elongational properties, Luo and Tanner [40] proposed an approach that assigns a different value of β to each mode ($\beta_{i}$). As multiple betas are not implemented in Ansys POLYFLOW, a best-fitted single value of β was employed despite the observed deviations.

**Figure A7:**
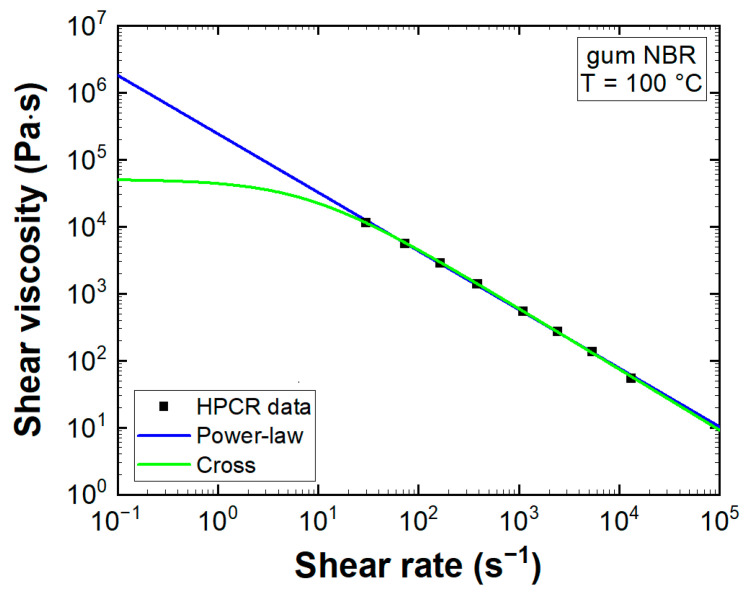
Steady-state shear viscosity of gum NBR compared to power-law and Cross model predictions.

Finally, coefficients (Table A4 and Table A5) of the two selected GNF models were determined applying the fitting tool implemented in Ansys POLYFLOW. Figure A7 proves the ability of both models to describe the steady-state shear viscosity of gum NBR well. However, the Cross model assumes a Newtonian plateau, resulting in differences between the two models in the lower shear rate region.

### 3.2. Numerical Modeling and Evaluation

The overall goal of this study was to test the ability of the K-BKZ/Wagner model to correctly predict pressure drops of an unfilled gum rubber in CFD simulation. Thus, four different capillary dies applied in HPCR tests with varying L/D ratios (0.2/1, 5/1, 10/1, 20/1) were modeled in Ansys POLYFLOW (Figure 1). At the inlet a fully developed normal velocity profile with a pre-defined volumetric flow rate *Q* was imposed (BC 1). Moreover, a normal and tangential velocity of zero was assigned at the capillary walls (BC 2). Next, use was made of the axisymmetric die geometry by applying a tangential force and normal velocity of zero at the axis of symmetry (BC 3). Since extruded strands of gum NBR clearly exhibited die swelling at all apparent shear rate levels (Figure A5), a “zero force” boundary condition (BC 4) was applied. This BC considered non-zero normal stresses at the outlet and, as a consequence, exit pressure dropped associated with die swelling.

**Figure 8:**
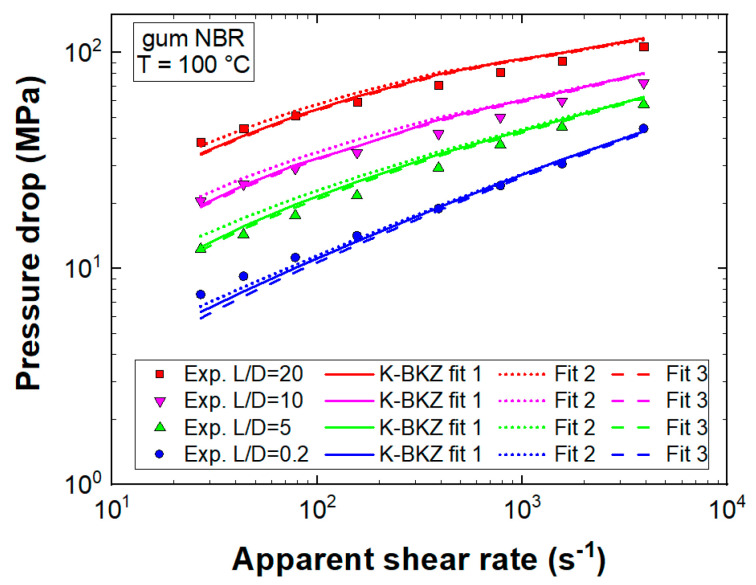
Measured pressure data compared to CFD simulation results; K-BKZ fit 1 represents fitted LVE data measured at F_n_ < 1 N (θ = 0), K-BKZ fit 2 represents fitted LVE data measured at F_n_ = 10 N (θ = 0), and K-BKZ fit 3 represents fitted LVE data measured at F_n_ < 1 N (θ = −0.15).

First, the influence of normal forces applied on the sample in SAOS testing (K-BKZ fit 2) as well as the influence of the model parameter θ (K-BKZ fit 3) were analyzed. For this purpose, Figure 8 compares recorded HPCR data with CFD simulation results, which proved the excellent pressure prediction accuracy of all three K-BKZ fits for gum NBR.

Mean deviations of K-BKZ fit 1, fit 2, and fit 3 to recorded pressure data were 8.6, 10.1, and 8.3%, respectively, indicating an insensitivity to the applied normal force and to the model parameter θ.

The tapered orifice (L/D = 0.2/1) represented an extreme condition, which was dominated by the contraction flow from the larger reservoir (∅ = 15 mm) in the small die (∅ = 1 mm). Thus, its measured pressure level was strongly affected by both entrance and exit pressure drops and consequently by the viscoelasticity of the material. The K-BKZ/Wagner model was able to correctly predict these extreme conditions for gum NBR with mean deviations (compared to recorded pressure drops of the L/D = 0.2/1 die) of 7.5, 5.9, and 9.3% for K-BKZ fit 1, fit 2, and fit 3, respectively. In contrast, pressure drops in the L/D = 20/1 die were dominated by the capillary flow and consequently by shear deformation. However, the K-BKZ model predicted also the second condition well with mean deviations (compared to recorded pressure drops of the L/D = 20/1 die) of 8.4, 7.6, and 8.0% for K-BKZ fit 1, fit 2, and fit 3, respectively. These results proved that the K-BKZ/Wagner was in fact able to correctly predict pressure drops of an unfilled gum rubber in both contraction and shear-dominated geometries. Thus, the observed deviations in our recent studies for two highly carbon black filled rubber compounds [25,26] can now be attributed to the inability of the K-BKZ/Wagner model to reflect the decreasing molecular mobility with increasing filler content. As a consequence, the K-BKZ/Wagner model is not applicable to highly filled polymer systems. The research groups of Walter Friesenbichler, Evan Mitsoulis, Ines Kühnert, and Sven Wießner have agreed to address this issue, aiming to develop a holistic viscoelastic–plastic constitutive rheological model for particle-filled, multiphase polymer systems based on the original K-BKZ equations.

**Figure 9:**
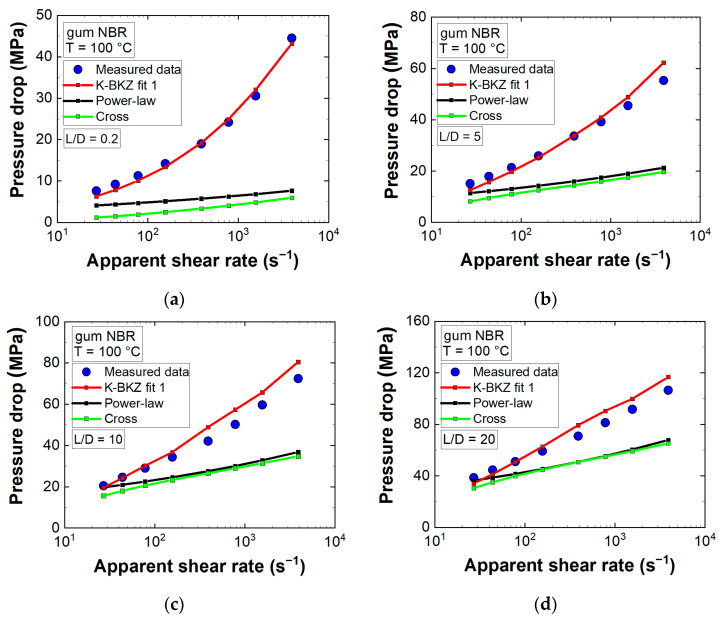
Measured pressure data compared to CFD simulation results of the (**a**) tapered orifice die (L/D = 0.2/1); (**b**) abrupt capillary die with a length-to-diameter (L/D) ratio of 5/1; (**c**) abrupt capillary die with L/D = 10/1; (**d**) abrupt capillary die with L/D = 20/1.

Finally, the influence of viscoelastic modeling was assessed by comparing CFD simulation results of K-BKZ fit 1 to pressure predictions of two generalized Newtonian fluid flow models (Figure 9).

Both GNF models (BCs listed in Table 1) underestimated measured pressure data distinctly. Deviations increased with rising apparent shear rate level and decreasing L/D ratio. Any GNF model considers viscous stresses only and intrinsically assumes a ratio of three between steady-state shear and uniaxial elongational viscosities. However, gum NBR exhibited a material response dominated by elasticity (Figure 4b), leading to an additional pressure drop at the exit and a ratio between η and $\eta_{e},$ clearly exceeding three (Figure 7), leading to an enhanced pressure level at the entrance. With decreasing L/D ratio these two factors became more and more important and mean deviations increased accordingly.

Moreover, Figure 7 displays an increasing ratio between η and $\eta_{e}$ with rising apparent shear rate level ($\eta_{e}/\eta_{\mathrm{Cross}}=10.4\left|\dot{\gamma },\dot{\varepsilon }=26{\;s}^{-1}\right.;{\;\eta }_{e}/\eta_{\mathrm{Cross}}=19\left|\dot{\gamma },\dot{\varepsilon }=207{\;s}^{-1}\right.$). Thus, the largest deviations between measured data and GNF predictions were expected and found to be true for the orifice die (L/D = 0.2) at the highest apparent shear rate level. Considering all dies as well as apparent shear rate levels, mean deviations to recorded pressure data were 8.6, 49, and 39.3% for K-BKZ, Cross, and power-law models, respectively (Figure 9). These values highlighted the importance of viscoelastic modeling to correctly predict pressure drops of an unfilled gum rubber especially in contraction flow-dominated geometries.

## 4. Summary and Conclusions

The present study tested the applicability of the viscoelastic K-BKZ/Wagner model in CFD simulations to correctly predict pressure drops of an unfilled gum acrylonitrile butadiene rubber (NBR). First, exhaustive rheological testing was performed in the linear viscoelastic (LVE) and non-LVE deformation range, including small amplitude oscillatory shear (SAOS) as well as high pressure capillary rheometer (HPCR) tests. The obtained material properties were subsequently used to fit the K-BKZ/Wagner and two generalized Newtonian fluid (GNF) flow models (Cross, power-law). Moreover, the influence of normal forces applied on the sample in SAOS testing as well as the relation of first and second normal stress differences to viscoelastic modeling and CFD simulations were analyzed by performing in total three K-BKZ/Wagner fits. Second, capillary dies applied in HPCR tests with varying length-to-diameter (L/D) ratios were modeled in Ansys POLYFLOW. Comparing CFD simulation results to recorded pressure data proved (i) an excellent pressure prediction accuracy for the K-BKZ/Wagner model and consequently its applicability to unfilled rubber materials; (ii) an insensitivity of the K-BKZ/Wagner model to the applied normal force in SAOS testing as well as to the relation of first and second normal stress differences, provided that damping parameters are fitted to steady-state rheological data; and (iii) the crucial importance of viscoelastic modeling for unfilled gum rubber materials, as both GNF flow models severely underestimated measured pressure data, especially in contraction flow-dominated geometries.

## Figures and Tables

**Table 1:** Boundary conditions (BCs) applied in CFD simulations.

BC	Description
BC 1	Fully developed velocity profile (inlet)
BC 2	Normal and tangential velocities are zero (no slip at the wall)
BC 3	Tangential force and normal velocity are zero (axis of symmetry)
BC 4	Normal force and tangential velocity are zero (viscous outlet)
BC 4	Normal and tangential forces are zero “zero force BC” (viscoelastic outlet)

## Data Availability

All data presented are only available after request from the corresponding author assuming a formal approval of our company partners.

## Appendix

**Table A1:** K-BKZ/Wagner model parameters (fit 1) for gum NBR at the reference temperature of 100 °C (θ = 0); Fit 1 represents fitted LVE data measured at F_n_ < 1 N.

Mode	$\lambda_{i}\;\left(s\right)$	$g_{i}\;\left(\mathrm{Pa}\right)$	$\alpha \;\left(/\right)$	$\beta \;\left(/\right)$
1	1.0000 × 10^−5^	1.6671 × 10^5^	0.1252	0.25
2	7.1969 × 10^−5^	1.6671 × 10^5^		
3	5.1795 × 10^−3^	1.6671 × 10^5^		
4	3.7276 × 10^−3^	1.6671 × 10^5^		
5	2.6827 × 10^−2^	1.6671 × 10^5^		
6	1.9307 × 10^−1^	7.4994 × 10^4^		
7	1.3895 × 10^−0^	2.9212 × 10^4^		
8	1.0000 × 10^1^	2.3313 × 10^4^		

**Table A2:** K-BKZ/Wagner model parameters (fit 2) for gum NBR at the reference temperature of 100 °C (θ = 0); Fit 2 represents fitted LVE data measured at F_n_ = 10 N.

Mode	$\lambda_{i}\;\left(s\right)$	$g_{i}\;\left(\mathrm{Pa}\right)$	$\alpha \;\left(/\right)$	$\beta \;\left(/\right)$
1	1.0000 × 10^−5^	2.3535 × 10^5^	0.1850	0.14
2	7.1969 × 10^−5^	2.3535 × 10^5^		
3	5.1795 × 10^−3^	2.3535 × 10^5^		
4	3.7276 × 10^−3^	2.3535 × 10^5^		
5	2.6827 × 10^−2^	2.3535 × 10^5^		
6	1.9307 × 10^−1^	1.0004 × 10^4^		
7	1.3895 × 10^0^	3.9518 × 10^4^		
8	1.0000 × 10^1^	3.0996 × 10^4^		

**Table A3:** K-BKZ/Wagner model parameters (fit 3) for gum NBR at the reference temperature of 100 °C (θ = –0.15); Fit 3 represents fitted LVE data measured at F_n_ < 1 N.

Mode	$\lambda_{i}\;\left(s\right)$	$g_{i}\;\left(\mathrm{Pa}\right)$	$\alpha \;\left(/\right)$	$\beta \;\left(/\right)$
1	1.0000 × 10^−5^	1.6671 × 10^5^	0.1260	0.20
2	7.1969 × 10^−5^	1.6671 × 10^5^		
3	5.1795 × 10^−3^	1.6671 × 10^5^		
4	3.7276 × 10^−3^	1.6671 × 10^5^		
5	2.6827 × 10^−2^	1.6671 × 10^5^		
6	1.9307 × 10^−1^	7.4994 × 10^4^		
7	1.3895 × 10^0^	2.9212 × 10^4^		
8	1.0000 × 10^1^	2.3313 × 10^4^		

**Table A4:** Cross model parameters for gum NBR at the reference temperature of 100 °C.

$\eta_{0}\left(\mathrm{Pa}\;\cdot \;s\right)$	$\lambda \;\left(s\right)$	$m\;\left(/\right)$
5.076 × 10^4^	1.31 × 10^−1^	9.095 × 10^−1^

**Table A5:** Power-law model parameters for gum NBR at the reference temperature of 100 °C.

$K\;\left(\mathrm{Pa}\;\cdot \;s\right)$	$\lambda \;\left(s\right)$	$n\;\left(/\right)$
8.467 × 10^2^	1.55 × 10^−3^	1.254 × 10^−1^
